# Genomic characterization, antimicrobial resistance, and virulence profiling of *Escherichia coli* isolated from diarrheic calves in Gansu, China

**DOI:** 10.3389/fmicb.2025.1729295

**Published:** 2026-01-06

**Authors:** Qi Meng, Liang Chang, Shengming Wang, Guopeng Lu

**Affiliations:** 1 Key Laboratory for Livestock and Poultry Disease Prevention and Control in Southeast Gansu, Gansu Institute of Animal Husbandry and Veterinary Medicine, Pingliang, China; 2 Zhuanglang County Animal Husbandry and Veterinary Center, Pingliang, China

**Keywords:** antimicrobial resistance, calf diarrhea, *Escherichia coli*, molecular epidemiology, virulence genes, whole-genome sequencing

## Abstract

**Introduction:**

This study provides a comprehensive genomic investigation of *Escherichia coli* isolated from diarrheic calves in Gansu Province, China, a region with significant livestock production.

**Methods:**

We employed whole-genome sequencing on 15 isolates from 15 different farms to characterize their molecular subtypes, plasmid repertoires, virulence gene profiles, and antibiotic resistance mechanisms.

**Results:**

Our analysis revealed high genetic diversity with 10 sequence types and 9 serotypes, including a novel serogroup. Phenotypic testing demonstrated widespread multidrug resistance, yet canonical resistance genes were absent in many resistant strains. Phylogenetic analysis elucidated the roles of both clonal dissemination and horizontal gene transfer.

**Discussion:**

These findings highlight the extensive genomic complexity of bovine *E. coli* in this region. The discrepancy between observed resistance and its genotypic basis underscores the need for integrated molecular surveillance. The small sample size limits generalizability, warranting confirmation in larger studies. This work situates its importance within the global “One Health” framework.

## 1 Introduction

Calf diarrhea is a prevalent cause of economic loss and compromised animal welfare in global cattle production (Torreggiani et al., 2025; Shoaib et al., 2023a; Zhang et al., 2025; Wang L. et al., 2024). Among the diverse etiological agents involved, pathogenic *Escherichia coli* is a primary bacterial cause of severe diarrheal outbreaks in calves, with its pathogenicity mediated by virulence factors such as adhesins, toxins, and iron acquisition systems (Azam et al., 2019; Shoaib et al., 2024a,2025a). The challenge is compounded by the rising prevalence of antimicrobial resistance (AMR) in bovine *E. coli*, particularly multidrug-resistant phenotypes (Shoaib et al., 2024b), which complicates treatment and may facilitate the spread of resistance genes (Saeed et al., 2023; Fernández et al., 2023).

The advent of whole-genome sequencing (WGS) has caused a major paradigm shift within the field of microbial genomics, offering unparalleled resolution for molecular epidemiology, phylogenetic analysis, and the dissection of pathogenic mechanisms (Tian et al., 2025). WGS has been demonstrated to exceed the capabilities of conventional typing methodologies by facilitating the high-fidelity characterization of sequence types (STs), serotypes, plasmid replicons, and the comprehensive profiling of virulence and antimicrobial resistance genes (ARGs) (Li et al., 2019). Moreover, comparative genomic analyses leveraging WGS data can delineate phylogenetic relationships, trace clonal dissemination, and illuminate the role of horizontal gene transfer (HGT) in the evolution and spread of virulent and resistant lineages (Meng et al., 2022; Li et al., 2025).

In China, although numerous studies have documented the prevalence and AMR profiles of bovine *E. coli* (Jia et al., 2024; Wang Z. et al., 2024; Ding et al., 2025), the genomic landscape of isolates from diarrheic calves in key livestock regions remains poorly elucidated. This is particularly true for Gansu Province, a significant cattle-farming region in Northwest China (Shoaib et al., 2023b). A critical knowledge gap exists regarding the population structure, genomic diversity, and the genetic basis of AMR and virulence in *E. coli* populations circulating within this region. A systematic genomic investigation is thus imperative to understand local pathogen dynamics, inform stewardship of antimicrobial use, and develop targeted control strategies.

To address this gap, we employed a WGS approach to conduct an in-depth genomic characterization of 15 *E. coli* isolates recovered from diarrheic calves across 15 distinct farms in Gansu Province. The specific objectives of this study were to: (i) determine their molecular subtypes (STs, serotypes and Clermont phylogroups); (ii) identify their plasmid repertoires and virulence gene profiles; (iii) characterize their resistome (ARG complement) and correlate it with phenotypic antimicrobial susceptibility; (iv) elucidate their phylogenetic relationships and population structure through core-genome SNP-based phylogeny, pangenome analysis, average nucleotide identity (ANI), and principal coordinates analysis (PCoA) based on Clusters of Orthologous Groups (COG) profiles; and (v) contextualize these isolates within the broader national epidemiology of bovine diarrheagenic *E. coli* by comparing them with publicly available genomes from other regions of China.

## 2 Materials and methods

### 2.1 Sample collection and processing

Fecal samples were collected from 15 severely diarrheic calves (aged 1–4 months), with one calf selected from each of 15 distinct farms in Gansu Province, China. The selected subjects represented the most severe clinical cases of diarrhea on each farm at the time of sampling, with all calves exhibiting hemorrhagic diarrhea, to increase the likelihood of isolating pathogenic *E. coli* strains (totally 72 calves with diarrhea). To capture the broad genetic diversity of *E. coli* within the region and avoid over-representation of a single clonal type, only one isolate was included from each farm. Any calves that had received antibiotic treatment were excluded from the study. Prior to sample collection, the perianal area was disinfected using 0.1% benzalkonium chloride. Subsequently, a fecal sample should be collected directly from the rectum using a sterile swab. Samples must be stored in individually packaged Carr-Blair transport medium (Hopebio, China) and transported at a temperature between 5 °C and 25 °C. Storage time should not exceed 24 h.

Samples were streaked onto eosin-methylene blue agar and incubated at 37 °C for 16 h. A single metallic-sheen colony per calf was selected, cultured in LB broth (Hopebio, China), and identified as *Escherichia coli* via 16S rRNA gene PCR. From each confirmed isolate, an aliquot was used for DNA extraction, and a glycerol stock was prepared for long-term storage at −80 °C.

### 2.2 Antibiotic susceptibility testing

The disk diffusion method was employed to test the isolated strains for susceptibility to ampicillin (10 μg), ceftriaxone (30 μg), cefepime (30 μg), cefoxitin (30 μg), meropenem (10 μg), gentamicin (10 μg), amikacin (30 μg), kanamycin (30 μg), chloramphenicol (30 μg), tetracycline (30 μg), doxycycline (30 μg), levofloxacin (10 μg), ofloxacin (5 μg), ciprofloxacin (5 μg), and Trimethoprim-sulfamethoxazole (1.25/23.75 μg). This method was selected in accordance with the antimicrobial susceptibility testing criteria established by the Clinical and Laboratory Standards Institute (CLSI). The quality control strain employed in this study was *Escherichia coli* ATCC 25922.

### 2.3 Whole-genome sequencing, assembly and annotation

Genomic DNA was extracted from overnight cultures of each *E. coli* isolate using a commercial kit (Tiangen, China). DNA quality and concentration were verified by agarose gel electrophoresis and NanoDrop spectrophotometry.

Sequencing libraries were prepared from fragmented DNA, and paired-end sequencing was performed on an Illumina HiSeq X platform (Illumina Inc., USA). After quality control and adapter trimming, the high-quality reads were assembled *de novo* using SPAdes v3.11.1. The resulting genomes were annotated using Prodigal v2.6.2.

### 2.4 Whole genome sequencing analysis

The sequence type (ST) of each isolate was identified using the MLST tool^1^ in conjunction with the PubMLST database. In order to ascertain the distribution of O and H serotypes, analysis was performed using the ECTyper.^2^ The phylogroup for each strain was identified *in silico* using the ClermonTyping.^3^ The virulence factor profiles of *E. coli* were identified using the VFDB^4^ and antibiotic resistance genes using CARD,^5^ ResFinder,^6^ MEGARes^7^ and ARG-ANNOT^8^ databases, respectively. The PointFinder^9^ was utilized for the purpose of detecting site mutations in antibiotic resistance genes within the chromosome. The minimum coverage and sequence similarity thresholds for screening were both set at 80%. Plasmid replicon types were identified using the PlasmidFinder v2.1.^10^ Utilizing *E. coli* K12 (Genebank Accession: GCA_000005845) as the reference genome, core single-nucleotide polymorphisms (SNPs) were generated with Snippy,^11^ and a maximum likelihood tree was constructed for 15 *E. coli* strains using FastTree v2.1.10^12^.

### 2.5 Comparative genomic analysis

To contextualize the isolates from this study within the broader population of *E. coli* associated with bovine diarrhea in China, a complete reference genome was retrieved from the National Center for Biotechnology Information (NCBI) database via the Bacterial and Viral Bioinformatics Resource Center (BV-BRC)^13^ in September 2025. The search and screening criteria were as follows: Initially, searches were conducted with the keywords “cattle” and “diarrhea,” employing “*Escherichia coli*” as the host species. Secondly, only domestic isolates for which complete genome sequencing and assembly were available were selected. Thirdly, in order to ensure data quality and comparability, only genomes with a completion status of “Complete” or “WGS” and a genome quality of “Good” were included. In order to provide a comprehensive reflection of the national epidemiological landscape, genomes originating from diverse geographic regions within China were included in the study. Phylogenetic analysis was performed using the Roary v3.13.0^14^ workflow to generate alignment sequences of the core genomes of isolated strains. Subsequently, the maximum likelihood phylogenetic tree was constructed using the FastTree v2.1.10 software, based on the GTR + CAT evolutionary model. The Average Nucleotide Identity analysis of *E. coli* was conducted using the FastANI.^15^ Principal coordinate analysis is predicated on COG functions of bacterial strains, utilizing the Bray-Curtis Faith distance algorithm.

## 3 Results

### 3.1 Whole genome sequencing of 15 *E. coli* strains

The assembly of each draft genome for the *E. coli* isolate was evaluated, and sequencing depth and number of contigs are reported in Supplementary Table 1. The Sequencing Depth for individual genomes was between 201X and 385X, and the number of contigs ranged from 51 to 94.

### 3.2 Whole genome sequencing analysis

The analysis of the sequences enabled the identification of nine distinct serotypes, including O86:H28 (*n* = 2), O75:H9 (*n* = 2), O5:H10 (*n* = 2), O115:H21 (*n* = 2), O9:H9 (*n* = 1), O88:H8 (*n* = 1), O117:H12 (*n* = 1) and O110:H2 (*n* = 1). One strain (Eco11) was classified as a novel serogroup, designated OgN31. A total of ten sequence types were identified among the analyzed isolates: ST58 (*n* = 4), ST156 (*n* = 2), ST43 (*n* = 2), and one isolate each of ST101, ST187, ST446, ST744, ST2522, ST3519, and ST12547. Phylogroup assignment revealed that 11 isolates (73.3%) belonged to phylogroup B1, while the remaining 4 isolates (26.7%) were classified as phylogroup A.

In the present study, a total of 15 *E. coli* isolates were subjected to a detailed plasmid replicon typing analysis, which yielded noteworthy results. The analysis revealed the presence of distinct plasmid carriage patterns, with a particular emphasis on the prevalence of IncF-type plasmids. Specifically, the IncFIB replicon was identified in 12 out of 15 strains, frequently in combination with IncFII (e.g., Eco2, Eco5, Eco6, Eco7, Eco12, Eco13) or IncFIC (e.g., Eco10, Eco14, Eco15). Strains such as Eco1 and Eco9 exhibited more diverse and complex replicon profiles, including IncHI2 and IncI1-I(Alpha). Conversely, Eco3 and Eco8 exhibited no detectable plasmids (Table 1).

**TABLE 1:** Plasmid replicon types identified in 15 *E. coli* strains from this study based on WGS analysis.

Strains	Plasmid replicon type
Eco1	IncFIB, IncHI2, IncI1-I(Alpha), IncQ1
Eco2	IncFIB, IncFII
Eco3	NA
Eco4	IncFII
Eco5	IncFIB, IncFII
Eco6	IncFIB, IncFII
Eco7	IncFIB, IncFII
Eco8	NA
Eco9	IncFIB, IncFII, IncHI2
Eco10	IncFIB, IncFIC
Eco11	IncFIA, IncFIB, IncFIC
Eco12	IncFIB, IncFII
Eco13	IncFIB, IncFII
Eco14	IncFIB, IncFIC
Eco15	IncFIB, IncFIC

**FIGURE 1:**
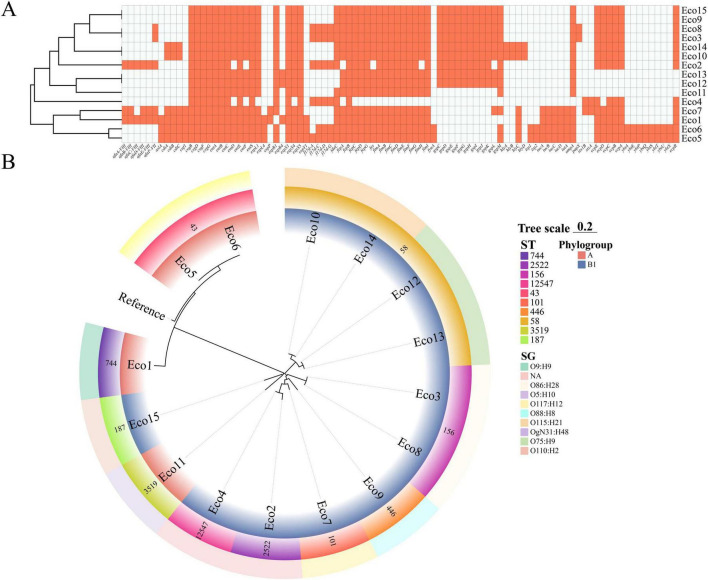
Genome analysis of 15 strains of *E. coli*. **(A)** The figure illustrates the virulence factors of 15 *E. coli* isolates. The presence of each virulence factor-encoding gene is indicated by an orange box (totally 92 virulence factors). The isolates were then hierarchically clustered based on their virulence factor profile using a method that employed the Euclidean metric distance with complete linkage clustering in columns. **(B)** A systematic genomic tree was constructed using the maximum likelihood method. This tree was based on core genome single nucleotide polymorphisms (cgSNPs) from 16 *E. coli* strains (15 *E. coli* strains from this study and one reference strain).

A comprehensive screening of virulence genes across 15 *E. coli* isolates Eco1–Eco15 was conducted, resulting in the observation of significant heterogeneity in virulence gene profiles (Supplementary Table 2). Isolates such as Eco1, Eco5, Eco6, and Eco7 exhibited a high abundance of virulence determinants, including genes associated with adhesion (e.g., *afa* cluster, *fim* genes), toxin production (e.g., *cdt*, *cnf1*, *hlyA*), and iron acquisition systems (e.g., *fyuA*, *irp2*, i*uc/iut*). In contrast, strains such as Eco11, Eco12, and Eco13 exhibited a more limited virulence repertoire, predominantly retaining only core colonization genes such as those involved in curli formation (*csg* operon) and enterobactin synthesis (*ent* genes). As depicted in the heat map (Figure 1A), a comparison of the virulence profiles of the strains under analysis is available.

### 3.3 Phylogenetic analysis

Based on the circular phylogenetic tree constructed from SNP data, the 15 *Escherichia coli* isolates are clearly segregated into 3 distinct clades, revealing strong phylogenetic signals consistent with their sequence types (STs) and serogroups (SGs) (Figure 1B). Isolates of ST58 (Eco10, Eco12, Eco13, Eco14) form a closely related cluster, further subdivided by serotypes O115:H21 and O75:H9, indicating microevolution within this lineage. Similarly, ST43 (Eco5, Eco6) and ST156 (Eco3, Eco8) each constitute monophyletic groups, corroborating their genetic relatedness. Notably, strains sharing the same ST and SG (e.g., Eco5 and Eco6: ST43, O5:H10) are positioned adjacently, underscoring the concordance between genomic background and surface antigen profile. All ST58, ST156, ST101, ST187, ST446, and ST2522 strains were identified as phylogroup B1, whereas ST43 and ST744 strains were assigned to phylogroup A.

### 3.4 Antibiotic sensitivity profile and resistance genes

**FIGURE 2:**
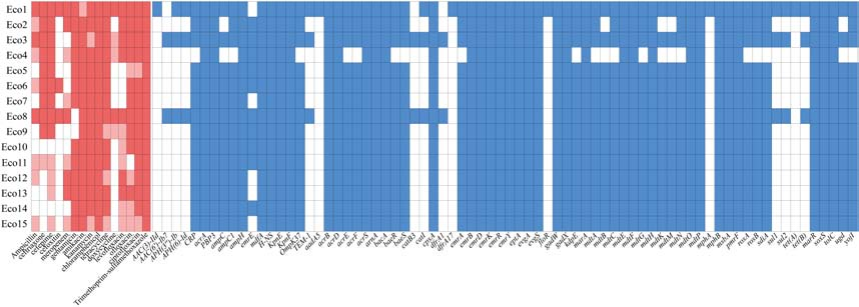
A heat map showing the antibiotic resistance profile and resistance genes of 15 *E. coli* strains. Within the context of the resistance spectrum, the color red is indicative of resistance, light red signifies intermediate, and white denotes susceptibility. In the heatmap showing the presence and absence of drug resistance genes, blue indicates presence, while white indicates absence.

A subsequent analysis of the antimicrobial resistance profiles indicated a high prevalence of multidrug resistance among bovine *E. coli* isolates. However, the genetic basis of this resistance was found to be more complex than initially assumed (Figure 2). While phenotypic resistance to sulfonamides was widespread, the canonical genes *sul1*, *sul2* were only detected in a limited number of strains (e.g., Eco1 and Eco3). Detection of mutations in the chromosomal drug resistance gene revealed mutations in *gyrA* and *parC* in the Eco1, Eco3 and Eco8 strains, as detailed in Supplementary Table 3. This finding suggests the probable existence of alternative, hitherto uncharacterized resistance mechanisms that are likely responsible for the predominant resistance phenotypes observed. Conversely, the presence of efflux pump genes (e.g., *acrAB-tolC*) was observed across all samples, indicating a fundamental contribution to the intrinsic resistance characteristics of this population. The ST43 lineage (Eco5, Eco6) demonstrated clonal dissemination of an identical, complex resistance profile, whereas ST58 strains exhibited marked heterogeneity, thereby underscoring the combined roles of clonal spread and horizontal gene transfer in shaping the AMR landscape.

### 3.5 Comparative genomic analysis

**FIGURE 3:**
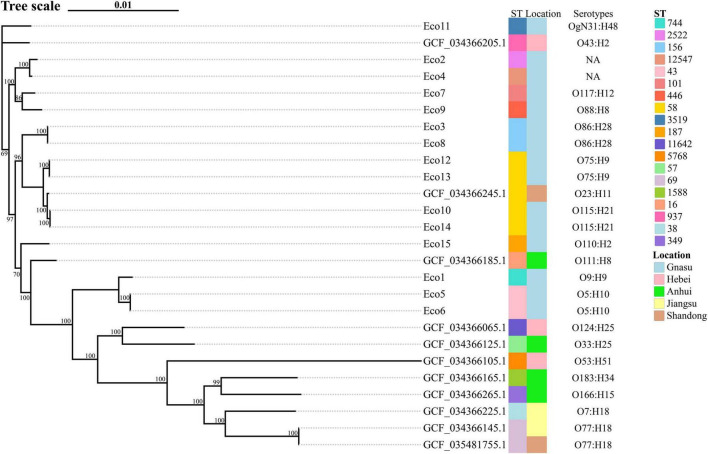
Phylogenetic and molecular characteristics of diarrheagenic *E. coli* isolates from cattle in China. The set includes 15 reference genomes from Gansu province strains and 11 additional reference genomes from cattle diarrheagenic *E. coli* strains from other Chinese provinces.

The pangenome-based phylogenetic analysis of *Escherichia coli* isolates from bovine diarrhea in China revealed that the population structure was primarily shaped by serotype, with distinct bovine-associated lineages identified (Supplementary Table 4). The study identified clinically relevant serotypes, including O5:H10 (Eco5, Eco6), O75:H9 (Eco12, Eco13), and O115:H21 (Eco10, Eco14), which formed well-supported, monophyletic clusters. These findings suggest the emergence and clonal dissemination of specific pathogenic lineages within Gansu China cattle. Conversely, the polyphyletic distribution of sequence types, such as ST58, suggests that this multi-locus genotype has been acquired independently by genetically distinct strains, likely through horizontal gene transfer of housekeeping genes within the bovine environment. It is noteworthy that the phylogenetic tree did not exhibit clear clustering by geographic origin, which could be indicative of potential inter-regional transmission events or the circulation of common ancestral lineages across China. The inclusion of Chinese reference genomes from diverse sources further contextualizes these clinical isolates within the broader population of bovine *E. coli* in China, highlighting the circulation of both common and unique serotypes associated with bovine diarrhea (Figure 3).

**FIGURE 4:**
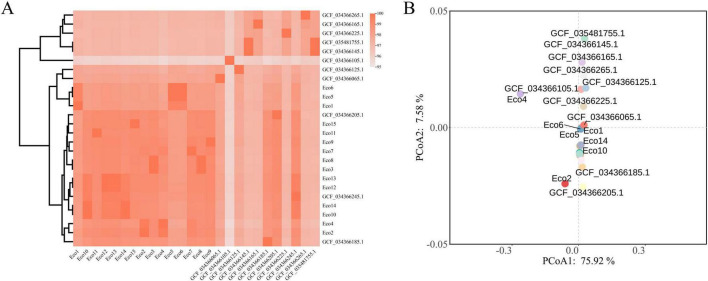
Principle coordinate analysis (PCoA) and Average Nucelotide Identity (ANI) analysis of 26 *E. coli* strains. **(A)** Whole-genome ANI analysis of 26 *E. coli* strains. **(B)** PCoA of the 26 *E. coli* strains based on COG function.

The ANI and COG-based PCoA analyses consistently revealed pronounced genomic divergence among the bovine diarrheagenic *E. coli* isolates, with clustering patterns largely concordant with serotypes and sequence types. In the ANI heatmap, isolates such as Eco5 and Eco6 (both ST43, O5:H10) exhibited high genomic similarity (ANI > 99%), forming a tight cluster with reference genomes, indicating clonal relatedness. Similarly, strains Eco12 and Eco13 (both ST58, O75:H9) showed high ANI values, supporting their close evolutionary relationship. In contrast, strains from polyphyletic STs (e.g., ST58 members Eco10, Eco14) displayed lower ANI with other ST58 isolates, confirming genetic heterogeneity within this lineage (Figure 4A). The PCoA based on COG functional profiles further supported this structuring, with the first principal coordinate (PCo1) explaining 75.92% of the variance, clearly separating strains into distinct functional groups. Notably, the clustering in PCoA correlated with both phylogenetic clades and virulence gene profiles, suggesting that functional differences in COG categories–particularly those involved in adhesion, toxin production, and iron acquisition–may underpin the ecological adaptation and pathogenic specialization of these lineages (Figure 4B).

## 4 Discussion

This study comprehensively characterized the genomes of 15 *Escherichia coli* strains isolated from calves suffering from diarrhea in Gansu Province, China, through whole-genome sequencing. The results obtained demonstrate that calf-derived *E. coli* in this region exhibit significant genetic diversity, multidrug resistance, and a complex virulence gene profile. This indicates the combined role of clonal transmission and horizontal gene transfer in shaping the evolution of this population. These findings align with recent international studies highlighting the genomic complexity and multidrug resistance of *E. coli* in diarrheic calves. For instance, a study from Egypt reported similar trends, where diarrheic calf-derived *E. coli* isolates exhibited a high prevalence of multidrug resistance and carried diverse virulence and antibiotic resistance genes, further underscoring the global nature of this challenge (Ezzat et al., 2023). The consistency between our results and those from geographically distinct regions reinforces the notion that clonal expansion and horizontal gene transfer are key drivers shaping the evolution of pathogenic *E. coli* in livestock populations worldwide.

The population structure was dominated by lineages with known pathogenic potential, such as ST58 and ST43. The ST58 strains exhibited considerable genetic and serotypic heterogeneity, supporting its role as a versatile lineage capable of acquiring diverse virulence and resistance traits through horizontal gene transfer, as observed elsewhere (Salinas et al., 2025; Yartey et al., 2025; Handal et al., 2025; de Lagarde et al., 2024). In contrast, the ST43 strains (Eco5, Eco6) were genetically identical, indicating local clonal dissemination on specific farms. Genome prediction of serogroup OgN31 further highlights the ongoing evolution and diversification of bacterial populations in this region.

Plasmid analysis highlighted the pivotal role of IncF-type plasmids (particularly IncFIB) in disseminating resistance and virulence genes, consistent with their notoriety in both animal and human pathogens (Shoaib et al., 2025b). The complex virulence profiles of strains like Eco1 and Eco5, carrying toxins (*cnf1*, *hlyC*) and the iuc/iut siderophore system, suggest a heightened potential for causing severe systemic infections (Nawaz et al., 2025).

A recognized limitation of this study is the use of disk diffusion method without concomitant MIC determination. The absence of MIC data may have limited our ability to detect subtle shifts in resistance levels and to correlate the degree of resistance with specific genetic mutations or the expression levels of resistance genes. This is particularly relevant given the observed discordance between widespread phenotypic resistance to sulfonamides and the limited detection of canonical resistance genes (*sul1*, *sul2*). It is also possible that uncharacterized or atypical resistance mechanisms–such as other gene variants, efflux pump overexpression, enzyme modification, or altered cell membrane permeability–may be the primary drivers of these phenotypes (Ramamurthy et al., 2022). Conversely, the ubiquitous presence of the *AcrAB-TolC* efflux pump system across all isolates constitutes the foundation of their intrinsic resistance (Nadi et al., 2024).

Comparative genomic analysis contextualized our isolates within the national landscape of bovine diarrheagenic *E. coli*. Phylogenetic tree revealed that clustering was primarily driven by serotype and ST rather than strict geographic origin. For instance, the close genetic relatedness between an O77:H18 (ST69) strain from Jiangsu and a reference from Shandong could be indicative of potential inter-regional transmission, though this requires confirmation with epidemiological data. The polyphyletic nature of ST58 further suggests that this sequence type has been independently acquired by genetically distinct backgrounds. Notably, the clustering in PCoA based on COG functional profiles aligned with both phylogenetic clades and virulence patterns. This suggests that genomic background dictates functional potential, and the acquisition of specific functional modules (including virulence-associated COGs) drives the ecological adaptation and pathogenic specialization of these lineages (Xue et al., 2024).

While this study provides valuable insights into the genomic landscape of *E. coli* from diarrheic calves in Gansu Province, several limitations must be considered when interpreting the results. First, the small sample size (*n* = 15) limits the statistical power to define the full scope of circulating clones and their prevalence. Larger-scale surveys are needed to confirm the emergence of lineages like ST43 and to track the dynamics of heterogeneous groups like ST58. Second, the disk method categorizes isolates as susceptible, intermediate, or resistant but does not provide the quantitative, continuous data that MICs offer. This limitation is particularly critical in light of our central finding of a genotype-phenotype discrepancy. MIC data could have revealed low-level or emerging resistance that falls into the “intermediate” category by disk diffusion, and could have helped correlate the degree of resistance with specific genetic mutations or the potential overexpression of efflux pumps. The absence of MICs thus hinders a more nuanced understanding of the resistance mechanisms at play. Third, the use of short-read sequencing alone prevented the complete assembly of plasmids and the determination of whether resistance and virulence genes were co-localized on the same mobile genetic elements. Long-read sequencing of key isolates is essential in future work to fully resolve these structures and understand the mechanisms of co-selection and transmission. Finally, as noted earlier, the phylogenetic inferences suggesting inter-regional transmission are based solely on genomic similarity. Without supporting epidemiological evidence, such as animal movement records or farm management data, these remain hypotheses to be tested. Genomic data can powerfully suggest transmission links, but it cannot confirm them (Sequeira et al., 2025).

## 5 Conclusion

In summary, this study represents the first systematic genomic analysis of *Escherichia coli* isolates from diarrheic calves in Gansu Province. Our findings reveal high genetic diversity and multidrug resistance among strains in this region, with IncF plasmids playing a pivotal role in transmitting resistance genes. We confirmed the local circulation of specific clones (e.g., ST43) and identified significant genetic heterogeneity within dominant lineages (e.g., ST58). Crucially, we observed mismatches between extensive phenotypic resistance and known genetic determinants, indicating substantial risks from unknown resistance mechanisms. These findings suggest that future control strategies should shift from simple pathogen identification to molecular surveillance of specific high-risk clones, and from predictions based solely on known genotypes to precision assessments that integrate both phenotypic and genotypic data. This study provides crucial genomic evidence for understanding and controlling the evolution and spread of pathogens in livestock within the “One Health” framework.

## Data Availability

The original contributions presented in the study are publicly available. This data can be found here: NCBI, accession number PRJNA1347210.

## Footnotes

1
https://github.com/tseemann/mlst

2
https://github.com/phac-nml/ecoli_serotyping

3
http://clermontyping.iame-research.center/

4
https://www.mgc.ac.cn/VFs/

5
https://card.mcmaster.ca/

6
https://bitbucket.org/genomicepidemiology/resfinder_db

7
https://db.meglab.org/

8
https://www.mediterranee-infection.com/acces-ressources/base-de-donnees/arg-annot-2/

9
https://bitbucket.org/genomicepidemiology/pointfinder

10
https://github.com/genomicepidemiology/plasmidfinder

11
https://github.com/tseemann/snippy

12
https://www.microbesonline.org/fasttree/

13
https://www.bv-brc.org/

14
https://sanger-pathogens.github.io/Roary/

15
https://github.com/ParBLiSS/FastANI
