# Supplementary material for: Genomic characterization, antimicrobial resistance, and virulence profiling of Escherichia coli isolated from diarrheic calves in Gansu, China
Source: Front Microbiol. 2026 Jan 6;16:1729295. doi: 10.3389/fmicb.2025.1729295 (PMC12816387; doi:10.3389/fmicb.2025.1729295)
Supplement: Supplementary file 1 [file Table_1.docx]

Supplementary Material

Table S1

Genomic Information of 15 *Escherichia coli* Isolates

| Isolates | Sequencing Depth | Number of contigs | Length of scaffold sequences | Coverage | Scaffold N50 | Counts of N50 |
| --- | --- | --- | --- | --- | --- | --- |
| Eco1 | 241X | 94 | 5123708 bp | 100% | 127925 | 11 |
| Eco2 | 283X | 94 | 4433319 bp | 100% | 57022 | 24 |
| Eco3 | 221X | 90 | 5076713 bp | 100% | 154902 | 9 |
| Eco4 | 385X | 94 | 3097626 bp | 100% | 34216 | 31 |
| Eco5 | 239X | 51 | 4759075 bp | 100% | 294418 | 6 |
| Eco6 | 232X | 66 | 4887369 bp | 100% | 234750 | 7 |
| Eco7 | 240X | 93 | 5020594 bp | 100% | 209916 | 9 |
| Eco8 | 234X | 86 | 5012398 bp | 100% | 168112 | 9 |
| Eco9 | 242X | 54 | 5051874 bp | 100% | 186695 | 8 |
| Eco10 | 230X | 94 | 5144797 bp | 100% | 177512 | 9 |
| Eco11 | 213X | 57 | 4663040 bp | 100% | 142074 | 11 |
| Eco12 | 380X | 59 | 4713976 bp | 100% | 271117 | 6 |
| Eco13 | 282X | 59 | 4713419 bp | 100% | 271117 | 6 |
| Eco14 | 203X | 94 | 5137670 bp | 100% | 177512 | 10 |
| Eco15 | 201X | 78 | 5004613 bp | 100% | 198500 | 9 |

Table S2

Virulence genes of 15 *Escherichia coli* Isolates

| Isolates | Virulence genes |
| --- | --- |
| Eco1 | *afaA-VIII,afaB-VIII,afaC-VIII,afaD-VIII,afaE-VIII,afaF-VII, aslA,cdtA,cdtB,cdtC,cnf1,csgB,csgD,csgF,csgG,entA,entB,entC,entD,entE,entF,entS,espL1,espL4,espP,espR4,espX1,espX4,espX5,espY1,fdeC,fepA,fepB,fepC,fepD,fepG,fes,fimA,fimB,fimC,fimD,fimE,fimF,fimG,fimH,fimI,hlyC,iucA,iucB,iucC,iucD,iutA,ompA,ecpE,ecpD,ecpC,ecpB,ecpA,ecpR* |
| Eco2 | *afaA-VIII,afaB-VIII,afaC-VIII,afaD-VIII,afaE-VIII,afaF-VII,csgB,csgD,csgF,csgG,entA,entB,entC,entE,entS,espL1,espR1,espX1,espX4,espX5,f17d-A,f17d-C,f17d-D,f17d-G,fdeC,fepB,fepC,fepD,fepG,fimA,fimB,fimC,fimD,fimE,fimF,fimG,fimI,gspC,gspD,gspE,gspF,gspG,gspH,gspI,gspJ,gspK,gspM,ompA,ecpE,ecpD,ecpC,ecpB,ecpA,ecpR* |
| Eco3 | *afaF-VII, csgB,csgD,csgF,csgG,entA,entB,entC,entD,entE,entF,entS,espL1,espR1,espX1,espX4,espX5,f17d-A,f17d-C,f17d-D,f17d-G, fdeC,fepA,fepB,fepC,fepD,fepG,fes,fimA,fimB,fimC,fimD,fimE,fimF,fimG,fimH,fimI,gspC,gspD,gspE,gspF,gspG,gspH,gspI,gspJ,gspK,gspL,gspM,ompA,papX,ecpE,ecpD,ecpC,ecpB,ecpA,ecpR* |
| Eco4 | *csgB,csgD,csgF,csgG,entA,entB,entC,entE,entS,espR1,espX,,espX5,f17d-A,f17d-C,f17d-D,f17d-G,fdeC,fepB,stx1B,stxA,ecpE,ecpC,ecpB,ecpA,ecpR* |
| Eco5 | *aslA,cdtA,cdtB,cdtC,cnf1,csgB,csgD,csgF,csgG,entA,entB,entC,entD,entE,entF,entS,espL1,espL4,espR1,espX1,espX4,espX5,espY1,f17d-A,f17d-C,f17d-D,f17d-G,fdeC,fepA,fepB,fepC,fepD,fepG,fes,fimA,fimB,fimC,fimD,fimE,fimF,fimG,fimH,fimI,fyuA,gspM,hlyC,irp1,irp2,iucA,iucB,iucC,iucD,iutA,ompA,ecpE,ecpD,ecpC,ecpB,ecpA,ybtA,ybtE,ybtP,ybtQ,ybtS,ybtT,ybtU,ybtX,ecpR* |
| Eco6 | *aslA,cdtA,cdtB,cdtC,cnf1,csgB,csgD,csgF,csgG,entA,entB,entC,entD,entE,entF,entS,espL1,espL4,espR1,espX1,espX4,espX5,espY1,f17d-C,f17d-D,f17d-G,fdeC,fepA,fepB,fepC,fepD,fepG,fes,fimA,fimB,fimC,fimD,fimE,fimF,fimG,fimH,fimI,fyuA,gspM,hlyC,irp1,irp2,iucA,iucB,iucC,iucD,iutA,ompA,ecpE,ecpD,ecpC,ecpB,ecpA,ybtA,ybtE,ybtP,ybtQ,ybtS,ybtT,ybtU,ybtX,ecpR* |
| Eco7 | *afaA-VIII,afaB-VIII,afaD-VIII,afaE-VIII,afaF-VII,cdtA,cdtB,cdtC,cnf1,csgB,csgD,csgF,csgG,entA,entB,entC,entD,entE,entF,entS,espL1,espP,espR1,espX1,espX4,espX5,fdeC,fepA,fepB,fepC,fepD,fepG,fes,fimA,fimB,fimC,fimD,fimE,fimF,fimG,fimH,fimI,gspL,gspM,hlyC,iucA,iucB,iucC,iucD,iutA,ompA,stx1B,stxA,ecpE,ecpD,ecpC,ecpB,ecpA,ecpR* |
| Eco8 | *afaF-VII,csgB,csgD,csgF,csgG,entA,entB,entC,entD,entE,entF,entS,espL1,espR1,espX1,espX4,espX5,f17d-A,f17d-C,f17d-D,f17d-G,fdeC,fepA,fepB,fepC,fepD,fepG,fes,fimA,fimB,fimC,fimD,fimE,fimF,fimG,fimH,fimI,gspC,gspD,gspE,gspF,gspG,gspH,gspI,gspJ,gspK,gspL,gspM,ompA,papX,ecpE,ecpD,ecpC,ecpB,ecpA,ecpR* |
| Eco9 | *csgB,csgD,csgF,csgG,entA,entB,entC,entD,entE,entF,entS,espL1,espR1,espX1,espX4,espX5,fdeC,fepA,fepB,fepC,fepD,fepG,fes,fimA,fimB,fimC,fimD,fimE,fimF,fimG,fimH,fimI,gspC,gspD,gspE,gspF,gspG,gspH,gspI,gspJ,gspK,gspL,gspM,ompA,ecpE,ecpD,ecpC,ecpB,ecpA,ecpR* |
| Eco10 | *cdtA,cdtB,cdtC,csgB,csgD,csgF,csgG,entA,entB,entC,entD,entE,entF,entS,espL1,espR1,espX1,espX4,espX5,fdeC,fepA,fepB,fepC,fepD,fepG,fes,fimA,fimB,fimC,fimD,fimE,fimF,fimG,fimH,fimI,gspC,gspD,gspE,gspF,gspG,gspH,gspI,gspJ,gspK,gspL,gspM,hlyA,hlyB,hlyC,hlyD,ompA,ecpE,ecpD,ecpC,ecpB,ecpA,ecpR* |
| Eco11 | *csgB,csgD,csgF,csgG,entA,entB,entC,entD,entE,entF,entS,espL1,espR1,espX1,espX4,espX5,fdeC,fepA,fepB,fepC,fepD,fepG,fes,fimA,fimB,fimC,fimD,fimE,fimF,fimG,fimH,fimI,gspM,ompA* |
| Eco12 | *csgB,csgD,csgF,csgG,entA,entB,entC,entD,entE,entF,entS,espL1,espR1,espR4,espX1,espX4,espX5,fepA,fepB,fepC,fepD,fepG,fes,fimA,fimB,fimC,fimD,fimE,fimF,fimG,fimHfimI,gspC,gspD,gspE,gspF,gspG,gspH,gspI,gspJ,gspK,gspL,gspM,ompA* |
| Eco13 | *csgB,csgD,csgF,csgG,entA,entB,entC,entD,entE,entF,entS,espL1,espR1,espR4,espX1,espX4,espX5,fepA,fepB,fepC,fepD,fepG,fes,fimA,fimB,fimC,fimD,fimE,fimF,fimG,fimH,fimI,gspC,gspD,gspE,gspF,gspG,gspH,gspI,gspJ,gspK,gspL,gspM,ompA* |
| Eco14 | *cdtA,cdtB,cdtC,csgB,csgD,csgF,csgG,entA,entB,entC,entD,entE,entF,entS,espL1,espR1,espX1,espX4,espX5,fdeC,fepA,fepB,fepC,fepD,fepG,fes,fimA,fimB,fimC,fimD,fimE,fimF,fimG,fimH,fimI,gspC,gspD,gspE,gspF,gspG,gspH,gspI,gspJ,gspK,gspL,gspM,hlyA,hlyB,hlyC,hlyD,ompA,ecpE,ecpD,ecpC,ecpB,ecpA,ecpR* |
| Eco15 | *csgB,csgD,csgF,csgG,entA,entB,entC,entD,entE,entF,entS,espL1,espR1,espX1,espX4,espX5,fdeC,fepA,fepB,fepC,fepD,fepG,fes,fimA,fimB,fimC,fimD,fimE,fimF,fimG,fimH,fimI,gspC,gspD,gspE,gspF,gspG,gspH,gspI,gspJ,gspK,gspL,gspM,ompA,ecpE,ecpD,ecpC,ecpB,ecpA,ecpR* |

Table S3

Antibiotic resistance gene mutations in the chromosome

| Isolate ID | Gene | Mutation | %Identity |
| --- | --- | --- | --- |
| Eco1 | *gyrA* | D87N, S83L | 99.92 |
|  | *parC* | A56T, S80I | 99.87 |
| Eco3 | *gyrA* | D87N, S83L | 99.24 |
|  | *parC* | E84G, S80I | 99.16 |
| Eco8 | *gyrA* | D87N, S83L | 99.24 |
|  | *parC* | E84G, S80I | 99.16 |

Table S4

Strains of Pangenome analysis

| Strains | ST | SG | Location | Year | Host |
| --- | --- | --- | --- | --- | --- |
| Eco1 | 744 | O9:H9 | Gnasu | 2025 | Cow |
| Eco2 | 2522 | NA | Gnasu | 2025 | Cow |
| Eco3 | 156 | O86:H28 | Gnasu | 2025 | Cow |
| Eco4 | 12547 | NA | Gnasu | 2025 | Cow |
| Eco5 | 43 | O5:H10 | Gnasu | 2025 | Cow |
| Eco6 | 43 | O5:H10 | Gnasu | 2025 | Cow |
| Eco7 | 101 | O117:H12 | Gnasu | 2025 | Cow |
| Eco8 | 156 | O86:H28 | Gnasu | 2025 | Cow |
| Eco9 | 446 | O88:H8 | Gnasu | 2025 | Cow |
| Eco10 | 58 | O115:H21 | Gnasu | 2025 | Cow |
| Eco11 | 3519 | OgN31:H48 | Gnasu | 2025 | Cow |
| Eco12 | 58 | O75:H9 | Gnasu | 2025 | Cow |
| Eco13 | 58 | O75:H9 | Gnasu | 2025 | Cow |
| Eco14 | 58 | O115:H21 | Gnasu | 2025 | Cow |
| Eco15 | 187 | O110:H2 | Gnasu | 2025 | Cow |
| GCF_034366065.1 | 11642 | O124:H25 | Hebei | 2020 | Cow |
| GCF_034366105.1 | 5768 | O53:H51 | Hebei | 2020 | Cow |
| GCF_034366125.1 | 57 | O33:H25 | Anhui | 2021 | Cow |
| GCF_034366145.1 | 69 | O77:H18 | Jiangsu | 2021 | Cow |
| GCF_034366165.1 | 1588 | O183:H34 | Anhui | 2021 | Cow |
| GCF_034366185.1 | 16 | O111:H8 | Anhui | 2021 | Cow |
| GCF_034366205.1 | 937 | O43:H2 | Hebei | 2020 | Cow |
| GCF_034366225.1 | 38 | O7:H18 | Jiangsu | 2020 | Cow |
| GCF_034366245.1 | 58 | O23:H11 | Shandong | 2023 | Cow |
| GCF_034366265.1 | 349 | O166:H15 | Anhui | 2021 | Cow |
| GCF_035481755.1 | 69 | O77:H18 | Shandong | 2023 | Cow |
